# Pulmonary fibrosis in a mouse model of sarcoid granulomatosis induced by booster challenge with *Propionibacterium acnes*

**DOI:** 10.18632/oncotarget.9397

**Published:** 2016-05-17

**Authors:** Dingyuan Jiang, Xiaoxi Huang, Jing Geng, Run Dong, Shuhong Li, Zheng Liu, Chen Wang, Huaping Dai

**Affiliations:** ^1^ Department of Respiratory and Critical Care Medicine, Beijing Key Laboratory of Respiratory and Pulmonary Circulation Disorders, Beijing Chao-Yang Hospital-Beijing Institute of Respiratory Medicine, Capital Medical University, Beijing, P.R. China; ^2^ Department of Medical Research, Beijing Chao-Yang Hospital, Capital Medical University, Beijing, P.R. China; ^3^ Department of Pulmonary and Critical Care Medicine, China-Japan Friendship Hospital, Beijing, P.R. China

**Keywords:** sarcoidosis, granuloma, inflammation, pulmonary fibrosis, mice, Pathology Section

## Abstract

Pulmonary fibrosis (PF) associated with chronic sarcoidosis remains poorly understood, and no experimental model is currently available for this condition. Previous studies have shown that *Propionibacterium acnes* (PA) was associated with sarcoidosis and induced granuloma formation in mice. Here, we investigated whether repeated challenge with PA induces persistent inflammation leading to sarcoidosis followed by PF in mice. Specifically, C57BL/6 mice were inoculated intraperitoneally and subjected to intratracheal challenge with PA, and then were booster-challenged with either PA or phosphate-buffered saline on day 28. Inflammation, granulomata, and features of fibrosis were evaluated every 7 days until day 70. Complete remission of lung granulomata was apparent on day 42 in the sarcoid-remission group. However, granulomata was present from days 21 to 70 in mice that received PA boosting. Inflammatory cell counts and Th1 cytokine levels in lung lavage fluids were elevated up to day 70. Furthermore, fibrotic changes in the lungs were observed around granulomatous and peribronchovascular regions after PA boosting. Taken together, these findings suggest that development of PF following sarcoidosis may result from continuous PA infection and inflammation. Repeated boosting with PA to induce PF might be a useful model for future studies of sarcoidosis-associated PF.

## INTRODUCTION

Pulmonary sarcoidosis is a common inflammatory lung disease that is characterized by accumulation of CD4+T cells, macrophages, and non-caseating granulomata in the lung [1, 2]. Its clinical outcomes are variable, as more than half of the affected patients achieve remission, whereas up to a third develop chronic disease. In chronic pulmonary sarcoidosis, pulmonary fibrosis is a major comorbidity and is associated with a higher risk of pulmonary hypertension, the need for lung transplantation, and increased mortality [3, 4]. Although the pathogenesis of sarcoidosis had been widely investigated, the underlying mechanisms whereby granulomatous inflammation progresses into chronic sarcoidosis with pulmonary fibrosis remain poorly characterized.

The clonality of CD4^+^ T cells that express distinct T-cell receptors in sarcoidosis [5] supports an antigen-induced etiology, and epidemiological studies have identified environmental and occupational risk factors for sarcoidosis [6]. Recently, *Propionibacterium acnes* (PA) was implicated as the etiological agent for sarcoidosis [7-9 ]. In classic studies, mice inoculated with PA were found to develop granulomatous inflammation [10,-12] similar to human sarcoidosis granulomata. However, these granulomatous pathological changes all showed a self-limiting course with spontaneous granulomata remission.

Sarcoid granulomatas are formed in response to a T-helper 1(Th1)-type immune response driven by antigenic stimulation, in which macrophages release a great variety of cytokines such as TNF-alpha and IL-12 [13, 14]. T-cell differentiation and plasticity are determined by the many factors produced during the inflammatory response including the coordinated actions of cytokines and chemokines [15]. Additionally, in chronic sarcoidosis, a transition from a Th1 to Th2 polarized response has been proposed. In the context of a Th2 cytokine milieu, macrophages might contribute to the development of fibrosis by producing TGF-β and CCL18 [16, 17]. Furthermore, the up-regulation of inflammation-associated genes in fibrotic sarcoidosis has been reported [18], and intense inflammation is thought to be predominantly associated with the progression to fibrotic sarcoidosis. Thus, the sarcoidosis associated with pulmonary fibrosis is considered to be an extension of granulomatous inflammation.

However, the specific mechanisms underlying the chronic sarcoidosis associated with fibrosis are less well characterized and lack an animal disease model. Additionally, much of the current understanding of the pathogenesis of sarcoidosis has focused on granulomatous inflammation. Microbe-induced host responses might promote the aggregation and persistence of granulomatous lesions [19], and an inability to clear antigens could result in chronic disease. Given the increasing interest in determining whether or not PA can act as a trigger of chronic lung inflammation, we investigated whether antigen administration could induce a state of chronic sarcoidosis that would ultimately result in pulmonary fibrosis following granulomatous inflammation.

## RESULTS

### The way of inoculation and challenge in mice with heat-killed PA

**Figure 1:**
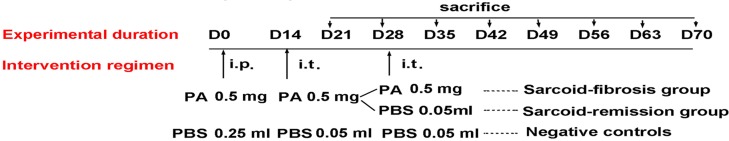
Treatment and experimental regimen in each group of mice i.t. indicates injection *via* the intratracheal route.

On day 0, 0.25 mL of the 2 mg/mL heat-killed PA suspension (a total of 0.5 mg) was injected intraperitoneally into mice. On day 14, mice were anesthetized with 1% sodium pentobarbital and challenged with 0.05 mL of the 10 mg/mL heat-killed PA suspension (a total of 0.5 mg) *via* the intratracheal route. PA inoculation and intratracheal challenge could induce sarcoid-granulomatosis in the lung. Sarcoidosis mice were given booster challenge on day 28 with another 0.05 mL of the 10 mg/mL heat-killed PA suspension (a total of 0.5 mg) intratracheally for a second challenge, these mice are sarcoid-fibrosis group. Giving 0.05 mL of sterile PBS on day 28 will show the natural disease course after once PA challenging on day 14, these mice were sarcoid-remission group. The mice were sacrificed at weekly intervals for 7 weeks after the final intratracheal injection. C57BL/6 mice that had been inoculated and challenged with sterile PBS (PBS/PBS/PBS) were used as negative controls for all experimental groups (Figure 1).

### Repeated boosting with PA induces chronic granulomata

**Figure 2:**
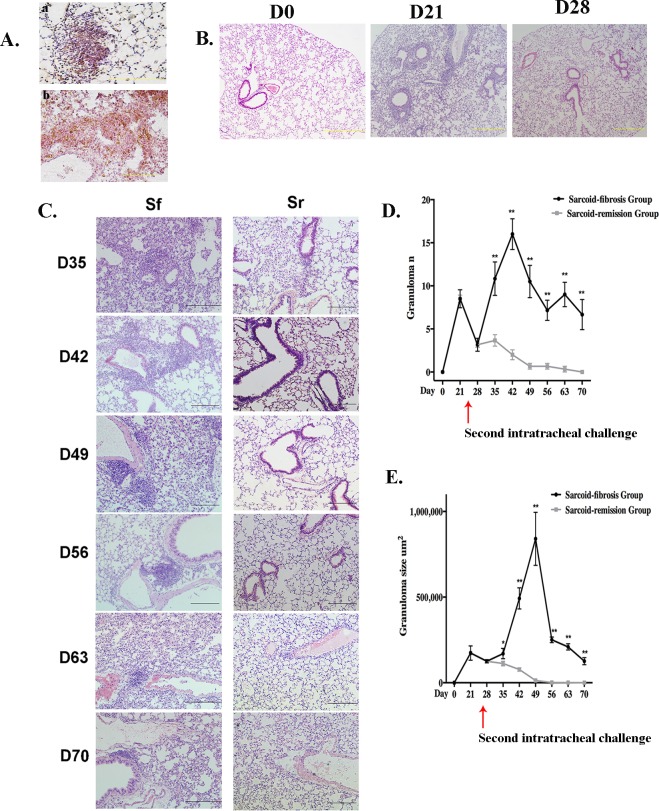
Repeated PA challenge induces chronic granulomatous inflammation **A.** Granulomata were composed of CD68^+^ (a) and CD4^+^ (b) cells identified by immunohistochemistry in paraffin-embedded lung tissue sections after the initial challenge with PA. Sections are shown at a magnification of ×40 ( scale bar = 50 μm). **B.** Granulomata and inflammation were prominent by day 21 and achieved remission by day 28. H&E stained sections are shown at magnifications of ×10 (scale bar = 200 μm) **C.** Lung histopathology for mice in the sarcoid-fibrosis (Sf) and sarcoid-remission (Sr) groups was evaluated at 7-day intervals. H&E stained sections are shown at magnifications of ×20 (scale bar = 100 μm). **D.**, **E.** The granuloma number (C) and size (D) as determined in the respective sections were compared between the sarcoid-fibrosis and sarcoid-remission mice every 7 days. In each group, six mice were analyzed at each time point; Data represent mean value±S.E.M *p < 0.05, **p < 0.01.

H&E-stained lung tissues from C57BL/6 mice at days 21 and 28 exhibited granulomata primarily in the pulmonary interstitium. The granulomata were composed of macrophages and CD4^+^ T cells (Figure 2A). Prominent granuloma formation and lymphohistocytic infiltrates were observed at day 21. Subsequently, granulomatous inflammation subsided, as less granulomata and inflammatory cell infiltration was observed on day 28 (Figure 2B). After the PBS challenge on day 28, granulomatous inflammation went into remission with no granuloma formation or peribronchovascular (PBV) infiltration observed at day 42. The lungs exhibited a mostly normal histological appearance by day 70 in the sarcoid-remission group (Figure 2C).

We next investigated whether or not repeated intratracheal challenge with heat-killed PA would exacerbate the inflammation or prolong the course of the pathology in the sarcoid-fibrotic group. Granulomata, lymphocytic PBV infiltration, and patchy pneumonia were observed on day 35. Lymphocyte infiltrates were denser and granulomata were more frequent, suggesting that granulomatous inflammation was induced by repeated PA challenge. On consecutive observation days, lymphocytic infiltration around the PBV and lung parenchyma was apparent and was defined by prominent histological inflammatory changes. However, granulomata were rarely detected on day 70, whereas the inflammatory infiltrate in the lung parenchyma persisted (Figure 2C). Both the number and size of the granulomata increased significantly after the second intratracheal PA challenge compared with the sarcoid-remission group (Figure 2D, 2E). Overall, repeated intratracheal challenge with PA induced a series of inflammatory events along with persistent granulomata and inflammatory histological changes.

### Pulmonary fibrosis following chronic granulomatous inflammation

**Figure 3:**
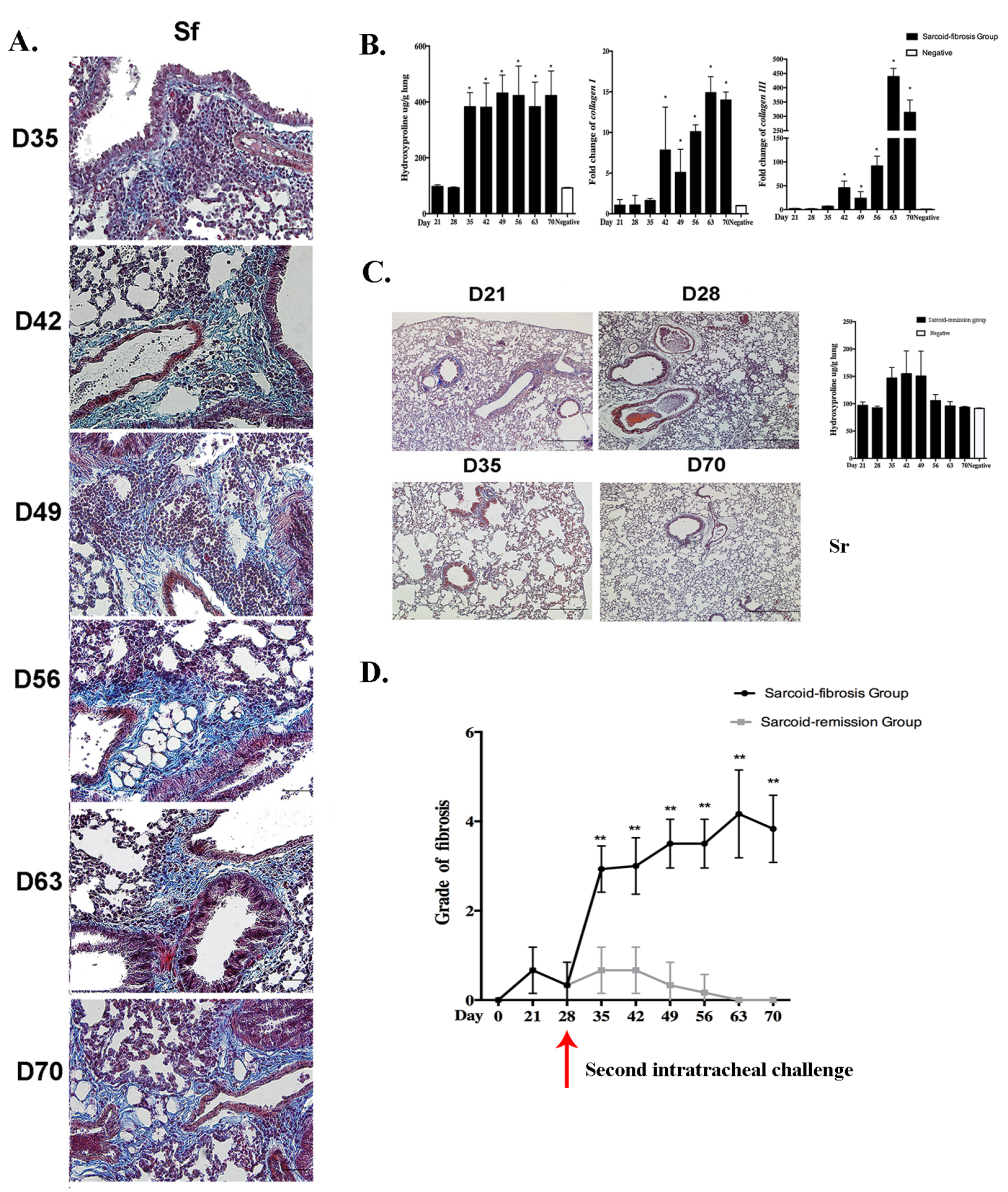
Repeated intratracheal PA challenge induces pulmonary fibrosis following granulomatous inflammation **A.** Histological analysis following Masson's trichrome staining demonstrated pulmonary fibrosis on days 35, 42, 49, 56, 63, and 70 in the sarcoid-fibrosis (Sf) group. Sections are shown at a magnification of ×40 (scale bar = 50 μm). **B.** Analysis of hydroxyproline compared with negative controls, together with mRNA transcript levels of collagen I and collagen III in sarcoid-fibrosis mice. **C.** No pulmonary fibrosis was detected on days 21, 28, 35, and 70 in the sarcoid-remission (Sr) group. Hydroxyproline analysis showed no differences between the sarcoid-remission group and the negative control. **D.** The grade of fibrosis was evaluated in the sarcoid-fibrosis and sarcoid-remission groups at 7-day intervals. Data represent the mean values±S.E.M.*p < 0.05, **p < 0.01.

Repeated intratracheal challenge with PA induced moderate to severe lymphocytic-rich inflammation along the bronchioles and vessels, sub-pleural areas, and parenchyma. These lesions were associated with significant fibrosis as indicated by Masson's trichrome staining of the lung tissues. Collagen deposition around the vessels, airways, and periphery of the granulomata was apparent on day 35. The distribution of collagen deposition was consistent with the inflammatory cell infiltration and was most prominent along the bronchovascular bundles. This parenchymal fibrotic change existed until day 70 (Figure 3A).

Lung tissues showed a significantly elevated hydroxyproline level compared with negative control groups, suggesting a higher composition of collagen. Real-time PCR analysis showed a significant increase in the mRNA expression of collagen I and III in the lung compared with the negative control group from days 42 to 70 (Figure 3B).

To verify the association between repeated PA administration and pulmonary fibrosis, Masson's trichrome staining was also performed in the sarcoid-remission group, which showed only a small amount of collagen around the bronchus and peripheral region of some granulomata on day 21. Pulmonary interstitial fibrosis was absent on days 21 to 70, and remission of inflammation was suggested by pathology and hydroxyproline assays (Figure 3C).

A comparison of the fibrotic score between the sarcoid-remission and sarcoid-fibrotic groups revealed significant differences after the second intratracheal challenge (Figure 3D), suggesting that repeated challenge with PA in mice could induce pulmonary fibrosis. Furthermore, the observed pulmonary fibrosis and granulomatous inflammation exhibited many similarities to the characteristic features of pulmonary fibrotic sarcoidosis seen in humans.

### Chronically increased numbers of inflammatory cells in the lung lavage fluid in the sarcoid-fibrotic group

**Figure 4:**
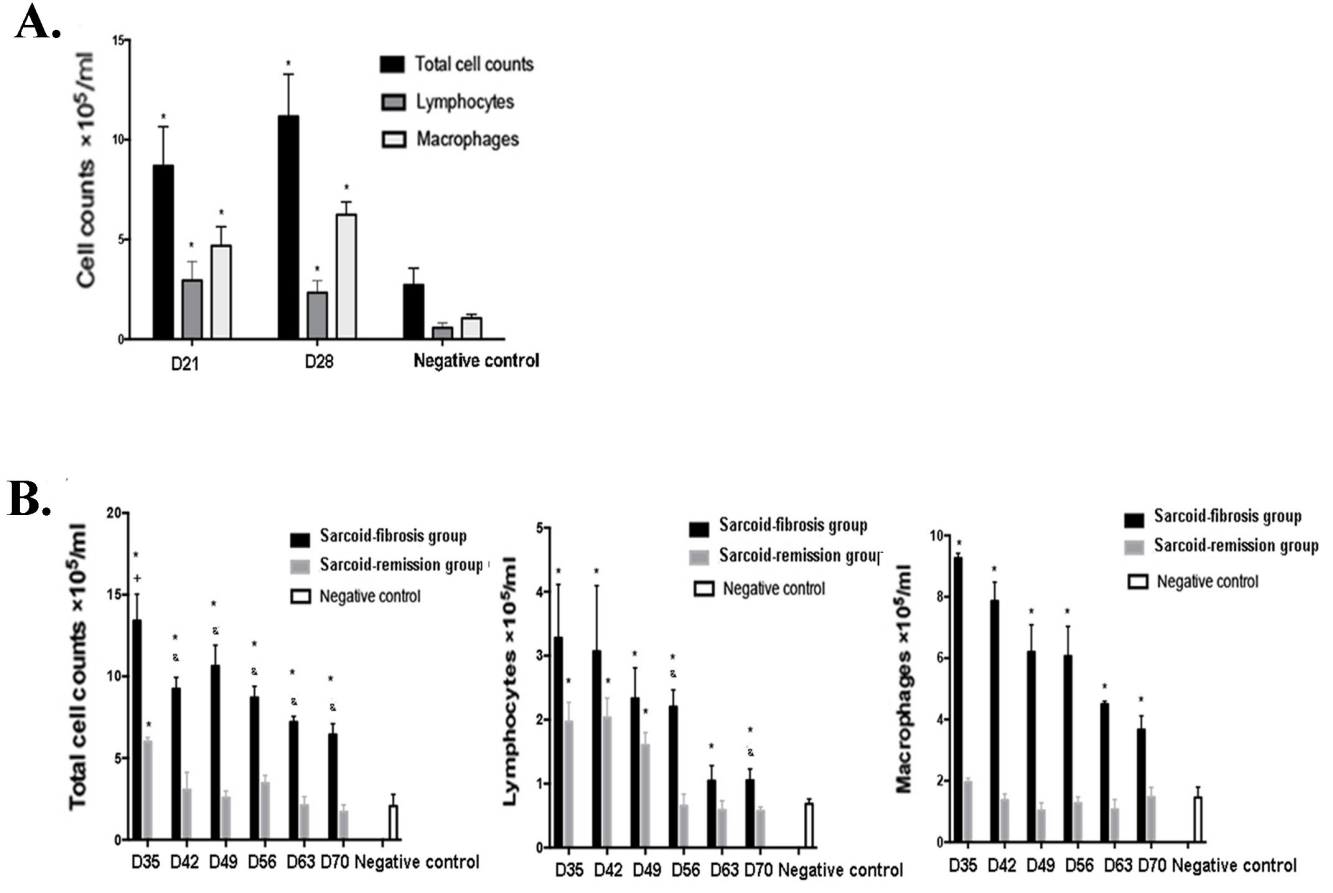
Increased inflammatory cell counts in the bronchoalveolar lavage fluid (BALF) of the sarcoid-fibrosis group mice **A.** More inflammatory cells were present in the BALF of sarcoidosis mice on days 21 and 28. **B.** Repeated PA challenge increased leukocyte recruitment to the lung; *p < 0.05 compared with the negative controls.

The composition of inflammatory cells in the lung lavage fluid was evaluated histologically, and increase in the total number of infiltrating cells was observed on days 21 and 28 compared with the negative controls. The numbers of lymphocytes and macrophages were also increased on days 21 and 28 (Figure 4A). After the challenge on day 28, the total cell counts in the sarcoid-remission group were elevated on day 35 and then returned to normal levels by day 42, but were maintained at a high level in the sarcoid-fibrotic group. Significant lymphocytosis developed in the sarcoid-fibrotic group by day 70. The number of macrophages was also increased in the sarcoid-fibrotic group. These findings show that repeated challenge with PA enhances the recruitment of inflammatory cells to the lung (Figure 4B).

### Elevated levels of proinflammatory cytokines and chemokines after booster challenge with PA

**Figure 5:**
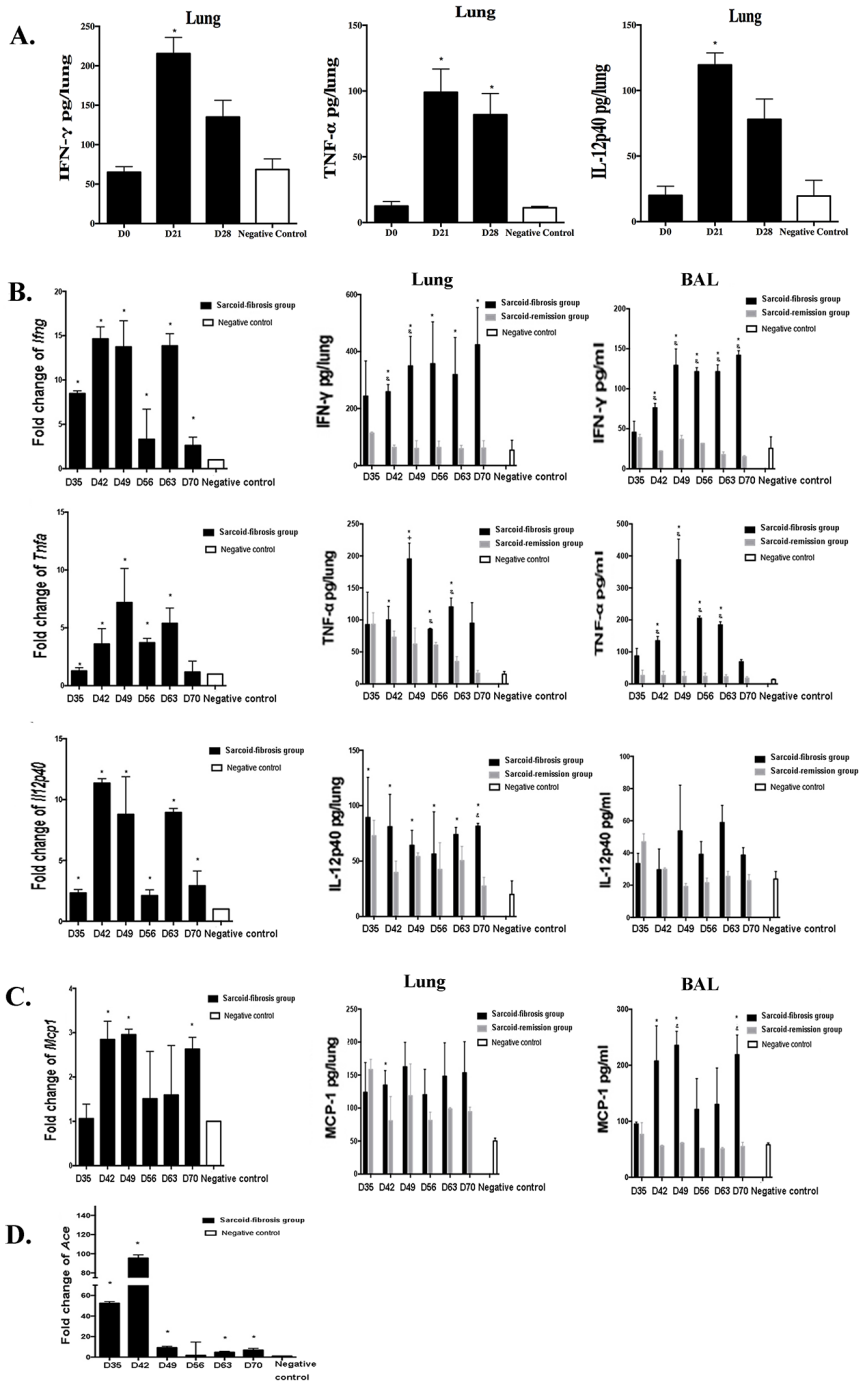
Increased levels of inflammatory cytokines in the lungs of the sarcoid-fibrosis group **A.** Inflammatory cytokines IFN-γ, TNF-α, and IL-12p40 in mouse lungs were elevated on day 21 after initial PA challenge. **B.** mRNA transcript levels of IFN-γ, TNF-α, and IL-12p40 in the lung along with increased protein levels of the respective gene products in lung homogenates and lavage fluid. **C.** mRNA transcript and protein levels of MCP-1 in the lung and BAL. **D.** *Ace* mRNA transcript levels in the lungs of the sarcoid-fibrosis group were analyzed. Data represent the mean values± S.E.M. *p < 0.05, compared with negative controls; &*p* < 0.05 compared with the sarcoid-remission group.

PA induced sarcoid-granulomatosis in the lungs of the mice after the initial challenge. Elevation of the Th1 immune response was detected along with granulomata formation on day 21 and day 28. IFN-γ, TNF-α, and IL-12P40 levels in the lungs were elevated on day 21 and day 28 before the second intratracheal challenge (Figure 5A).

After observing the sarcoid-granulomatosis inflammation, we determined the functional significance of the Th1-biased immune response after booster challenge from day 35. Cytokine expression in the lung was detected at both the mRNA and protein levels. Lung homogenates and lavage fluid showed a significant increase of *Ifng* mRNA levels on day 42 (Figure 5B). TNF-α protein levels in lung homogenates were significantly higher in sarcoid-granulomatosis mice than in sarcoid-remission mice from day 42 to day 63. No additional elevation of TNF-α levels was observed in the sarcoid-remission group compared with the negative controls after day 35 (Figure 5B). IL-12p40 levels in the lung homogenates of sarcoid-fibrotic mice were significantly elevated from days 35 to 70 compared with the negative controls, which showed only modest increases in the lung lavage fluid (Figure 5B).

MCP-1 is a C-C chemokine that attracts monocytes and macrophages to sites of inflammation. Significant up-regulation of *Mcp1* mRNA was detected on days 42, 49, and 70; MCP-1 protein levels were also elevated and showed a similar trend to that observed in the lung lavage fluid. In contrast, the sarcoid-remission group showed no changes in expression after day 35 (Figure 5C). These data indicate chronic inflammation in the sarcoid-fibrosis group.

### Elevated ACE expression in sarcoid-fibrotic mice

ACE levels are considered to indicate active disease in patients with sarcoidosis. *Ace* mRNA expression was significantly increased following repeated PA challenge compared with that in negative control mice (Figure 5D), indicating ongoing granulomatous inflammation.

### TGF-β1 is elevated in mice with pulmonary fibrosis

**Figure 6:**
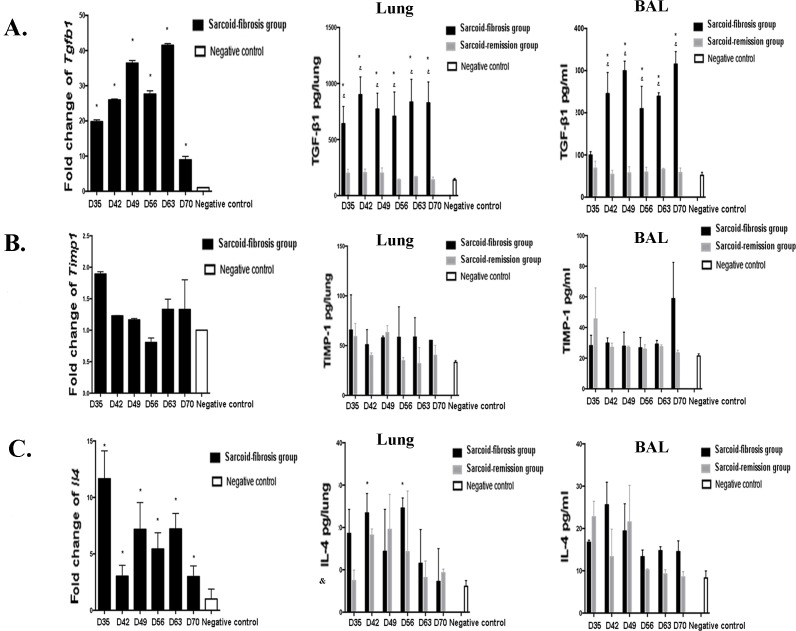
Expression of profibrotic cytokines in mice The mRNA transcript levels in the lung and protein levels in the lung homogenates and lavage fluid were measured for **A.** TGF-β1, **B.** TIMP-1, and **C.** IL-4. Data represent mean values±S.E.M. *p < 0.05 compared with negative controls; &*p* < 0.05 compared with the sarcoid-remission group.

To investigate the possible mechanisms that might be involved in the induction of pulmonary fibrosis associated with granulomatous inflammation, we measured the mRNA expression of TGF-β1, TIMP-1, and IL-4, which are thought to be involved in induction of pulmonary fibrosis, during the fibrotic stage in sarcoid-fibrotic mice. A significant increase in mRNA levels of TGF-β1 was observed from day 35 compared with the negative controls. TGF-β1 protein levels were continuously elevated through day 70 in the lung homogenates and lavage fluids (Figure 6A). In contrast, TIMP-1 protein levels were not different between repeatedly PA-exposed mice and negative control mice, although a two-fold increase in mRNA levels of TIMP-1 was detected on days 28 and 35 (Figure 6B). In addition, *Il4* mRNA transcript levels were significantly increased from day 35 to 70; however, the protein levels of IL-4 only increased on days 42 and 56 in the lung homogenates (Figure 6C).

Together, these data show that pulmonary fibrosis following granulomatous inflammation was induced by repeated PA challenge and persistent inflammation in mice.

## DISCUSSION

In this study, we induced chronic granulomatosis in the lungs of mice that lasted for 7 weeks after booster intratracheal injection of heat-killed PA. Compared with mice challenged only once with PA through the trachea, histopathological analysis revealed that the granuloma formation existed for a longer period of time in the booster-challenged animals. Furthermore, Masson's staining showed evidence that lung fibrosis developed following the granulomata after PA boosting, and the duration of inflammatory cell recruitment in the lung lavage fluid was also increased. Additionally, TNF-α levels were elevated after repeated PA challenge, and increased levels of TGF-β1 were observed during the lung fibrosis stage. Together, our findings indicate that repeated etiological challenge can induce granulomatous inflammation and its progression into lung fibrosis.

In previous mouse models of sarcoidosis granulomata induced by PA, PA was injected several times into the skin or footpad [20-22]. These models demonstrated granulomata in the lungs, skin, and liver. Subsequent cytokine detection confirmed a Th1-biased inflammatory response. These findings are similar to those observed in sarcoidosis in humans. In our present model, the disease features of the sarcoidosis on days 21 and 28, prior to PA boosting, were similar to those previously reported. However, the previous models showed a granulomatous course without fibrosis, thus mimicking the acute phase of sarcoidosis with granulomatous inflammation. In contrast, an animal model exhibiting chronic sarcoidosis such as that described in this study might provide mechanistic information regarding the progression of granulomatous inflammation into pulmonary fibrosis, which is urgently needed. Pulmonary fibrosis is observed in approximately 30% of patients with chronic sarcoidosis. The fibrosis following sarcoidosis exhibits unique characteristics that differ from those of idiopathic pulmonary fibrosis (IPF). Patients with end-stage sarcoidosis are characterized by fibrotic changes that are distributed in parallel with the granulomata and generally occur with the same lymphatic distribution as the inflammatory response [23, 24]. In contrast, fibroblastic foci and the dense collagen typical of IPF are rarely observed in fibrosis following sarcoidosis [25]. In bleomycin-induced fibrosis in mice, the fibrosis increases after acute lung injury and inflammation of deteriorated alveoli. The collagen is diffusely distributed in the interstitium in the lungs, and this fibrosis is associated with mortality, with most patients dying within in a month. In the present study, lung fibrosis appeared 14 days after PA boosting and was always accompanied by granulomata. Histological analysis revealed that fibrotic changes originated around the periphery of the granulomata collagen deposition was mostly around the bronchovascular bundles. Pulmonary fibrosis following granulomata occurred later and was milder compared to bleomycin-induced fibrosis. Notably, the granulomata induced by booster PA challenge in the animals was gradually resolved. Similarly, in late-stage sarcoid fibrosis in humans, granulomata might no longer be observable [26]. Thus, our findings revealed specific pulmonary fibrosis in combination with granulomata following repeated PA challenge, wherein the lung fibrosis appeared to be similar to that observed in sarcoidosis, but not in IPF.

It remains unclear whether or not progressive fibrosis results from persistent granulomatous inflammation [27]. In the sarcoid-remission group of the present study, no lung fibrosis was observed after the initial PA challenge. However, after boosting with PA, high levels of proinflammatory cytokines such as TNF-α and IFN-γ were observed along with the lung fibrosis. IFN-γ plays a pivotal role in granulomatous inflammation; however, it may also be involved in fibrosis. In previous studies, T cell activation with release of proinflammatory cytokines such as IFN-γ and TNF-α is suggested contribute to fibrosis in the heart and kidney [28], and levels of profibrotic cytokines such as TGF-β1 and IL-1 are increased in supernatants from activated T cells [29]. Previous studies showed that fibrotic sarcoidosis was associated with activation of an immune response, which was different from the pathogenesis of IPF [30]. In our study, booster challenge with PA induced activation of the Th1 immune response including expression of IFN-γ and TNF-α, which may have induced fibrosis and also activation of TGF-β1. BALF lymphocytes and ACE expression are both important in clinical assessments of sarcoidosis [5, 27]. In the present study, the number of inflammatory cells, especially lymphocytes, in the BALF increased from days 21 to 70. High levels of Ace mRNA transcripts were also detected in PA-boosted mice. These data suggest that persistent active disease occurred after booster PA challenge. Repeated PA boosting appears to cause persistent inflammation and immune activation, which might be important for the induction of fibrosis.

TGF-β1 is considered to play an important role in sarcoidosis-associated lung fibrosis, as previous studies [14, 31] identified associations between TGF-β polymorphisms and sarcoidosis. Therefore, in the present study we measured TGF-β1 levels in our experimental mice. Markedly high *Tgfb1* mRNA transcript levels were detected in sarcoid mice with lung fibrosis, suggesting that increased levels of TGF-β1 might play a role in the late stages of fibrosis induction. TGF-β1 has been established to play an important role in collagen secretion and fibroblast proliferation [32]. Accordingly, in our model, we found that collagen I and collagen III mRNA transcripts were up-regulated during the fibrotic process.

In contrast, there was no significant sign of a Th2-polarized immune response or the Th1-to-Th2 transition typically expected in sarcoidosis-associated fibrosis. It possibly due to the inoculated antigen, PA, reportedly enhances Th1 immune responses and inhibits the expansion of Th2 cells [33]. Th2 responses can be closely associated with fibrotic diseases including IPF [34], but have not been clearly linked to sarcoidosis-associated pulmonary fibrosis.

Further studies of pulmonary fibrosis following sarcoidosis are needed to elucidate the distinguishing pathological mechanisms. Studies to identify the associations between the onset of inflammation and the subsequent development of pulmonary fibrosis are also needed. In summary, we have developed a disease model that exhibits features similar to those of chronic sarcoidosis with pulmonary fibrosis in humans. Our study provides insights into the relationship between granulomatous inflammation and lung fibrosis. This work establishes a potential chronic sarcoidosis mouse model with pulmonary fibrosis, which might be a useful tool for determining the mechanisms of pulmonary fibrotic sarcoidosis and identifying potential therapeutic approaches.

## MATERIALS AND METHODS

### Ethics statement

The investigation was conducted in accordance with the ethical standards of the Declaration of Helsinki and according to national and international guidelines, and it was approved by the authors' institutional review board.

### Mice

Specific pathogen-free (SPF) 8- to 10-week-old C57BL/6 female mice were purchased from the Animal Center of Peking University (Beijing, China). All experimental and control mice were weight-matched, and their weight ranged from 20 to 25 g. The mice were housed under SPF conditions in the animal care facility of the Medical Research Center of Beijing Chaoyang Hospital. The mice were maintained on a chow diet in a 12-h light/12-h dark environment at 25°C and monitored with respect to their general state, fur condition, activity, and weight according to institutional guidelines. The mice were sacrificed at each observational time point by acceptable euthanasia guidelines using intraperitoneal pentobarbital overdose. This study was performed in strict accordance with the National Institutes of Health guide for the care and use of laboratory animals and approved by the Animal Care and Utilization Committee of Capital Medical University (AEEI-2014-034).

### Bacteria

PA were obtained from the American Type Cultures Collection (Manassas, VA, USA; ATCC #6919) and cultivated anaerobically in the microbiology laboratory at the China Center of Industrial Culture Collection. Cultures were used for experiments when the colonies became confluent. PA colonies were washed twice with phosphate-buffered saline (PBS) and then suspended in PBS. The PA suspension was heat-killed by autoclaving at 121°C for 20 min and then maintained at −80°C prior to use.

### Whole lung lavage and homogenates

Mice were euthanized with 50 mg/mL sodium pentobarbital (0.6 mg/10 g weight) according to published guidelines [35]. The collection of lung lavage fluid was as previously described [36]. Cell pellets were suspended in 1 mL of PBS to obtain total cell counts in a cytometry chamber. Then, cell pellets that had been resuspended in PBS were cytospun onto glass slides; these cells were stained with hematoxylin & eosin (H&E) solution, and 200 cells were counted for each sample for cell classification.

Whole lung tissues were homogenized in cold lysis buffer (PBS containing 0.1% Triton-X100 with 0.1% protease inhibitor) immediately after mouse dissection. Homogenates were centrifuged at 1500×*g* for 15 min at 4°C. Supernatants were stored at −80°C for cytokine analysis.

### Lung histology

The collection of mouse lungs for histology was performed as previously described [10]. Briefly, the lung was dehydrated, paraffin-embedded, and cut into 4-μm sections. Lung sections were stained with H&E and Masson's trichrome stain for assessment of pathological changes. Immunostaining was perfomed using antibodies against CD68 (Abcam, Cambridge, USA; 1:100) and CD4 (eBioscience, San Diego, CA; 1:100).

### Assessment of granuloma formation and fibrosis

The size and number of pulmonary granulomata were assessed in H&E-stained tissue sections. The severity of the pulmonary fibrosis was semi-quantitatively assessed according to Ashcroft's scoring system [37]. Sections were evaluated using an Olympus IX-51 microscope (Olympus, Tokyo, Japan). The area of total granulomata was calculated using ImagePro Plus software, version 6.0, by tracing individual borders.

### Hydroxyproline assay

Pulmonary levels of hydroxyproline were measured using conventional methods as previously described [34]. The levels of pulmonary hydroxyproline in each mouse were calculated based upon the lung weight, and the data are expressed as micrograms of hydroxyproline in each gram of lung tissue.

### ELISA

Concentrations of TNF-α, IFN-γ, IL-12p40, MCP-1, IL-4, TIMP-1, and TGF-β1 in the lung homogenates and lavage fluid were measured using ELISA kits purchased from eBioscience according to the manufacturer's instructions.

### RNA isolation and quantitative real-time polymerase chain reaction (PCR) analysis

Total RNA was extracted from one right lung lobe of each mouse using TRIzol reagent (Invitrogen, Carlsbad, CA, USA) according to the manufacturer's instructions. Then, cDNA was synthesized with oligo (dT) primers using a First-Strand cDNA Synthesis Kit (Tiangen, Beijing, China). The levels of cDNA were analyzed by quantitative PCR analysis using a SYBR Green real-time PCR kit (Tiangen) and an ABI PRISM 7500 detection system (Applied Biosystems, Foster City, CA, USA). DNA was amplified under the following cycling conditions: denaturation at 95°C for 15 s, annealing at 58°C for 20 s, and extension at 72°C for 20 s. Pre-mixed primers were purchased from Invitrogen to detect the mRNA transcript levels of *Tnfa*, *Ifng*, *Il12p40*, *Mcp1*, angiotensin converting enzyme (*Ace*), *Tgfβ1*, *Timp1*, *Il4*, *Col3a*, and *Col1*. β-actin was used as an internal control, and levels of each transcript were normalized to β-actin mRNA expression using the ΔΔCt method, with the experimental value for the negative control mice set to 1. Data are presented as the fold change over the expression in the lungs obtained from the negative control group.

### Statistical analysis

Groups of four to six mice were used for each time point in each experiment. All data are expressed as the mean ± SEM. All *in vitro* experiments were conducted with three independent replications. GraphPad Prism 6 (GraphPad, La Jolla, CA, USA) was used for statistical analyses using Student's *t*-test or one-way analysis of variance; *p* < 0.05 was considered to indicate statistically significant differences in all comparisons.
